# A rare presentation of adult colonic intussusception secondary to a descending colon lipoma: A case report

**DOI:** 10.1016/j.ijscr.2025.112119

**Published:** 2025-10-27

**Authors:** Biniam E. Zelelew, Dereje G. Andargie, Chernet T. Mengistie, Biruk T. Mengistie, Anteneh Gadisa, Abel G. Wubie

**Affiliations:** a University of Global Health Equity, Butaro, Rwanda; b Department of Surgery, College of Medicine and Health Science, University of Rwanda, Kigali, Rwanda; c School of Medicine, College of Health Sciences, Addis Ababa University, Addis Ababa, Ethiopia; d Department of Surgery, College of Medicine and Health Sciences, Bahir Dar University, Bahir Dar, Ethiopia

**Keywords:** Intussusception, Colon, Lipoma, Colonic neoplasms, Colectomy

## Abstract

**Introduction and importance:**

Adult colonic intussusception is a rare and often diagnostically challenging condition, typically associated with a pathological lead point. While malignant tumors are more common causes in adults, benign lesions like colonic lipomas can also be responsible, albeit infrequently.

**Case presentation:**

We report a case of a 54-year-old woman presenting with intermittent left lower abdominal pain and recent rectal bleeding. Contrast-enhanced abdominal CT demonstrated an incomplete descending colon intussusception with a fat-density mass as the lead point. She underwent a left hemicolectomy. Histopathology revealed a 4.2 × 4.3 cm pedunculated submucosal lipoma with no malignancy. Her postoperative course was uneventful, and she made a full recovery.

**Clinical discussion:**

Adult intussusception often requires surgical intervention due to the high likelihood of underlying malignancy. Preoperative imaging, particularly CT, plays a crucial role in diagnosis and identifying the nature of the lead point. In this case, a benign lipoma caused the intussusception, but surgical resection was still warranted to exclude malignancy and relieve symptoms.

**Conclusion:**

Though rare, colonic lipomas should be considered in adult intussusception cases. Cross-sectional imaging is essential for diagnosis, and surgical resection remains the mainstay of treatment. Early recognition and intervention can lead to excellent outcomes, as demonstrated in this case.

## Highlights

• Adult colonic intussusception is a rare clinical condition often caused by a malignant lead point.
• Large colonic lipomas are uncommon but can act as benign lead points for intussusception.
• CT imaging plays a central role in diagnosis, often revealing the characteristic “target sign” and fat-density mass.
• Surgical resection without prior reduction remains the standard treatment to rule out malignancy and ensure complete removal.

## 1 Introduction

Intussusception occurs when one segment of the intestine telescopes into an adjacent segment. In children, intussusception is relatively common and usually idiopathic, but adult intussusception is rare [1,2]. Indeed, adults account for less than 5 % of all intussusception cases and only about 1 %–5 % of adult bowel obstructions [1,3]. The incidence in adults is estimated at 2–3 cases per million population per year [4], making it an uncommon clinical entity. The distribution of intussusception sites varies as well: small bowel (enteroenteric) types predominate, while true colonic intussusception represents roughly 17–20 % of adult cases [5,6].

Unlike pediatric cases, nearly 80–90 % of adult intussusceptions have an identifiable pathological lead point [1,7]. These lead points are often neoplastic: malignant tumors are particularly common in colonic intussusceptions, whereas benign lesions (such as lipomas or polyps) more frequently underlie small intestinal intussusceptions [5,8]. A recent meta-analysis found that nearly half of colonic cases were due to malignant tumors [6]. Because of this, management generally follows oncologic resection without prior reduction [6,9].

Benign tumors can also lead to colonic intussusception, and colonic lipomas are an important example. Lipomas are benign submucosal adipose tumors that occur in 0.2–4.4 % of colonic lesions [10]. They are typically small and asymptomatic, often discovered incidentally during colonoscopy, surgery, or autopsy [10,11]. When large (>2 cm), colonic lipomas may cause symptoms such as pain, bleeding, or obstruction [11]. In rare cases, a large pedunculated lipoma can act as a lead point and cause colo-colonic intussusception. In a systematic review, colonic lipoma-induced intussusception was more common in middle-aged patients (40–70 years) and often presented with chronic or intermittent pain [10]. Associated symptoms can include rectal bleeding in about 16 % of cases and altered bowel habits in up to 18 %. Physical examination may be unremarkable in many patients [10,12].

Preoperative diagnosis relies on imaging. Contrast-enhanced CT is the modality of choice for suspected adult intussusception, revealing the characteristic “target” or “bowel-within-bowel” appearance [2,13]. CT can also often suggest the nature of the lead point: a lipoma appears as a well-defined fat-attenuation mass within the intussuscepted segment [13]. Colonoscopy may directly visualize a colonic lipoma (sometimes showing the “pillow sign”) but is not used for reduction and generally deferred when intussusception is suspected [10]. Given the high rate of malignancy in adult colonic intussusception, elective surgical resection is indicated once the diagnosis is made [3,9]. We present a case of descending colon intussusception from a pedunculated lipoma in a middle-aged woman, emphasizing the diagnostic approach and surgical management in light of the literature. This manuscript was prepared following the SCARE Guidelines 2025 criteria [21].

## 2 Case presentation

A 54-year-old woman presented with intermittent, stabbing pain in the left hypogastric and left flank regions, exacerbated by defecation. She had noted a bout of hematochezia several days before the pain began and had been treated empirically for amebiasis. She denied abdominal distension, fevers, weight loss, altered bowel habits beyond the hematochezia episode, and had no pulmonary or other systemic symptoms. There was no personal or family history of colorectal lesions or malignancy. She was a nonsmoker and did not use illicit drugs. She had a body mass index (BMI) of 23.6 kg/m^2^ (within the normal range) and no history of dyslipidemia, diabetes, or hypertension.

On examination, she was afebrile with normal vital signs and appeared comfortable. Abdominal exam showed mild, deep tenderness in the left lower quadrant without peritoneal signs. No masses were palpable, and the rest of the exam was unremarkable. Laboratory studies (complete blood count, metabolic panel) were within normal limits.

**Fig. 1:**
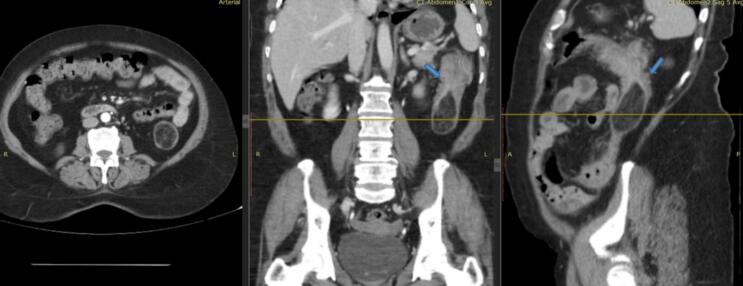
Contrast-enhanced abdominal CT images (axial, coronal, sagittal) showing incomplete intussusception of the descending colon with a well-circumscribed intraluminal fat-density mass (pedunculated lipoma) acting as the lead point (arrows).

A contrast-enhanced abdominal CT scan revealed an incomplete colo-colonic intussusception in the left (descending) colon. Within the intussuscepted segment was a well-circumscribed fat-density lesion measuring about 3.1 × 3.2 × 5.0 cm (anteroposterior × transverse × craniocaudal) (Fig. 1). The lesion acted as the lead point for partial telescoping of the bowel. There was no evidence of high-grade obstruction; proximal bowel loops were not significantly distended. Mild adjacent mucosal thickening was seen, but there were no signs of perforation or pericolic fat stranding. No lesions suggestive of metastases were seen in the liver, omentum, or peritoneum. A few small firm lymph nodes were noted along the left colic vessel pedicle. The preoperative impression was incomplete intussusception of the mid-descending colon, likely due to a pedunculated lipoma.

The patient was taken for an exploratory laparotomy under general anesthesia and placed in the supine position. A midline incision was made to enter the peritoneal cavity. Exploration revealed a segment of descending colon with partial intussusception caused by an intraluminal mass. A standard left hemicolectomy was performed via a lateral-to-medial mobilization, beginning with incision of the white line of Toldt to mobilize the descending colon. The left colic vessels were identified, ligated, and divided at their origin. Segmental resection of the involved colon, including the intussuscepted portion, was carried out en bloc. Bowel continuity was restored with a colo-colic end-to-end anastomosis using continuous 2/0 Vicryl sutures in a single layer. Hemostasis was secured, and the abdomen was closed in layers. No other intra-abdominal pathology was identified, and estimated blood loss was minimal. The specimen was subsequently opened on the back table, revealing a pedunculated submucosal mass on the antimesenteric border consistent with a lipoma.

**Fig. 2:**
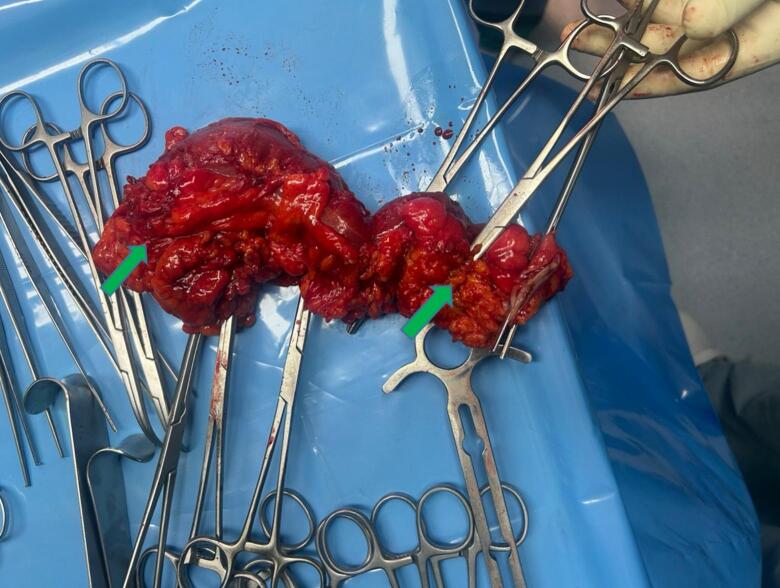
Gross photograph of the opened resected descending colon after left hemicolectomy. A pedunculated submucosal lipoma arising from the antimesenteric border, the lead point of the intussusception, is indicated by the arrows; the pedicle at the base of the lesion is visible. The mass measured 4.2 × 4.3 cm on gross examination. Surgical margins are shown and were free of tumor on histologic examination.

Gross examination of the specimen revealed a pedunculated submucosal mass measuring 4.2 × 4.3 cm on the antimesenteric border of the colon (Fig. 2). The intussusception involved this segment. Histopathology confirmed a benign lipoma composed of mature adipose tissue, with overlying intact mucosa and no dysplasia or malignancy. Surgical margins were free of tumor.

Postoperatively, the patient did well. Bowel function returned on postoperative day 2 (she passed flatus and stool), and oral feeding was initiated at that time. By day 5, she was tolerating a regular diet and ambulating fully, so she was discharged home in good condition. Follow-up at 2 and 4 weeks revealed complete resolution of symptoms and a return to normal activities without complications.

## 3 Discussion

Adult colonic intussusception is rare and often has a pathologic cause. Our patient's presentation of Intermittent left-sided pain and recent bleeding is similar to other reported cases of colonic lipoma-induced intussusception [9,10]. The commonest site of colonic lipomas is the ascending colon (45 %), followed by the sigmoid (30.3 %), descending (15.2 %), and transverse colon (9.1 %) [14]. In a review of colonic lipoma intussusception, abdominal pain was the predominant symptom (reported in ~83 % of cases), often intermittent or crampy, as in our patient [12]. Rectal bleeding or hematochezia is less common (~16 % of cases), but it occurred here and initially mimicked benign causes of bleeding. Notably, many patients have normal physical exams despite symptoms [10,12]; our patient had only mild localized tenderness without a palpable mass, which aligns with the nonspecific findings reported.

Imaging led to the diagnosis. Contrast-enhanced CT is the diagnostic modality of choice in adult intussusception, identifying the classic target appearance in over 70 % of cases [13]. On barium enema, lipomas may show fluctuation in size and shape during compression maneuvers, the so-called “squeeze sign”, which can support a benign lipomatous diagnosis [15]. In this patient, the CT not only confirmed intussusception but also identified a well-defined fat-attenuation mass as the lead point. Fat density lesions on CT strongly suggest a lipoma (as confirmed by this tumor's measured Hounsfield units) [16]. The CT also showed that the intussusception was incomplete and without high-grade obstruction or strangulation. Colonoscopy can sometimes visualize a lipoma (and even reduce intussusception in selected benign cases), but this is generally avoided in colonic intussusception due to the risk of perforation and the high likelihood of malignancy [3,10]. In our case, the combination of CT findings and lack of perforation guided an urgent but planned surgical approach.

The usual management of adult colo-colonic intussusception is surgical resection, given the high incidence of malignancy [9]. In our review, about half of large bowel intussusceptions are caused by cancer [6]. Even when imaging suggests a benign lipoma, definitive histology is needed. Thus, we performed a segmental colectomy without attempting reduction, consistent with oncologic principles [17]. The choice of left hemicolectomy was based on the location (mid-descending colon) and standard vascular anatomy. Literature on lipoma-induced intussusception notes that segmental resection or left colectomy is commonly employed for descending colon lesions [3,9,10]. Although minimally invasive (laparoscopic) approaches have been reported, we used open surgery because laparoscopic facilities were not available at our institution at the time of treatment; both approaches generally have good outcomes.

Histopathology confirmed the diagnosis of a benign pedunculated lipoma, as expected. Colonic lipomas arise from the submucosa and contain mature fat; malignant change is virtually unheard of [18]. The absence of malignancy in our patient is encouraging, but does not diminish the need for resection, since preoperative distinction is unreliable [10,19]. The patient's rapid postoperative recovery with discharge on day 5 and full return to activity is consistent with reports that surgical resection for lipoma intussusception usually carries an excellent prognosis [4,20].

This case highlights several teaching points. First, although rare, colonic lipoma should be in the differential for adult colonic intussusception, especially with a fatty CT lead point. Second, CT imaging is critical for diagnosis, revealing both intussusception and likely etiology. Finally, given the substantial risk of occult malignancy, colonic intussusceptions in adults warrant prompt surgical resection following oncologic principles [8,10,17]. In benign cases like this, patients typically do very well with complete resolution of symptoms.

## 4 Conclusion

Colonic intussusception in adults is a rare entity, most often secondary to a neoplastic lead point. This case of descending colon intussusception from a pedunculated lipoma illustrates that benign lesions may mimic obstruction or bleeding. Contrast-enhanced CT facilitated preoperative diagnosis by revealing both the intussusception and a fat-density lead mass. Definitive treatment was surgical resection without reduction, reflecting standard practice in adults, given the risk of malignancy. Histopathology confirmed a large benign lipoma, and the patient recovered uneventfully.

## Author contribution

**Biniam E. Zelelew:** Writing – review & editing, Visualization, Supervision.

**Dereje G. Andargie:** Conceptualization and Writing – Original Draft.

**Chernet T. Mengistie**: Writing – Original Draft, Resources, Data curation.

**Biruk T. Mengistie:** Writing – Original Draft, Visualization, Writing – review & editing.

**Anteneh Gadisa:** Visualization, Supervision.

**Abel G. Wubie:** Writing – review & editing, Data curation.

## Consent for publication

Written informed consent was obtained from the patient for publication of this case report and any accompanying images. A copy of the written consent is available for review by the Editor-in-Chief of this journal.

## Ethical approval

Ethical approval for this study (Protocol Number 5044/2022, with assigned number 008) was provided by the Ethical Committee of the University of Global Health Equity, Butaro, Rwanda, on 2 November 2023.

## Guarantor

Biniam E. Zelelew.

Dereje G. Andargie.

Chernet T. Mengistie.

## Research registration number

N/A.

## Funding

The authors didn't receive any specific funding for this work.

## Conflict of interest statement

The authors declare that they have no known competing financial interests or personal relationships that could have influenced the work reported in this paper.

## Data Availability

The data underlying the results presented in this work are available within the manuscript.
